# Pathology

**Published:** 1879-02

**Authors:** 


### PATHOLOGY.

THE POISON OF SYPHILIS.—Klebs. (*Centralbl. f. Chir.* No. 50, 1878.)

At the last congress of German naturalists, Klebs detailed his experiments made to detect the nature of syphilitic poison. If pieces of a Hunterian chancre are placed into a solution of gelatine, a vegetation of fungi is speedily observed. This consists of minute rods, at first of lively mobility, which array themselves subsequently in oval masses. The cultivation of this fungus was continued by transferring some germs into fresh solutions of gelatine. Small quantities of this turbid fluid were placed underneath the skin of a monkey. After six weeks the animal suffered from indurated ulcers in the mouth of a decidedly syphilitic appearance. The autopsy revealed, besides, syphilitic alterations of the dura mater and of the lungs. In another monkey similar syphilitic appearances were caused by direct inoculation with a portion of a Hunterian chancre. Different tissues and especially the blood of these animals placed into solutions of gelatine gave rise again to the above described fungus. Klebs here claims, that this fungi constitute the syphilitic virus, and proposes to call them helico-monads.

